# Infections with *Chlamydia pneumoniae* and SARS-CoV-2 and Alzheimer’s disease pathogenesis

**DOI:** 10.3389/fnagi.2025.1587782

**Published:** 2025-06-13

**Authors:** Alexa Romanella, Maegan McCall, Rachel Corwin, Alaha Abdul Faruq, Emily Lingo, Sanya Bhambhani, Christine J. Hammond, Brian J. Balin

**Affiliations:** ^1^Department of Bio-Medical Sciences, Center for Chronic Disorders of Aging, Philadelphia College of Osteopathic Medicine (PCOM), Philadelphia, PA, United States; ^2^Division of Research, Center for Chronic Disorders of Aging, Philadelphia College of Osteopathic Medicine (PCOM), Philadelphia, PA, United States; ^3^Division of Research, Center for Chronic Disorders of Aging, Alzheimer’s Pathobiome Initiative, Philadelphia College of Osteopathic Medicine (PCOM), Philadelphia, PA, United States; ^4^Department of Bio-Medical Sciences, Center for Chronic Disorders of Aging, Alzheimer’s Pathobiome Initiative, Philadelphia College of Osteopathic Medicine (PCOM), Philadelphia, PA, United States

**Keywords:** Alzheimer’s disease, neuroinflammation, *Chlamydia pneumoniae*, SARS-CoV-2, olfaction, blood-brain barrier

## Abstract

**Introduction:**

Alzheimer’s disease (AD) is the most prevalent neurodegenerative disease in the world, but our understanding of causation is still lacking. A current evidence-based hypothesis proposes that certain infectious agents initiate the neurodegeneration consistent with AD. Two infectious agents correlated to AD pathogenesis are *Chlamydia pneumoniae* (Cpn), a respiratory obligate intracellular bacterium, and severe acute respiratory syndrome coronavirus 2 (SARS-CoV-2), the coronavirus responsible for the COVID-19 pandemic. Both organisms may predispose susceptible populations to disease manifestations, such as AD.

**Methods:**

This review focused on peer-reviewed original research and review articles evaluating the potential association of Cpn and SARS-CoV-2 with AD. Our focus included: genetic risk with expression of APOEε4 and other biomarkers common to AD including interleukin-6 (IL-6), chemokine ligand 2 (CCL2), neuropilin-1 (NRP1), and structural/functional aspects of the infectious processes and resultant neuroinflammation.

**Results:**

Both Cpn and SARS-CoV-2 may infect the neuroepithelium of the olfactory system to enter the brain. Cpn binds to heparan sulfate proteoglycans for entry into mucosal cells. SARS-CoV-2 infects epithelia after binding to ACE2 receptors. Once inside the neuroepithelium, the pathogens may traffic to the olfactory bulbs. NRP1, an abundant receptor in AD, also potentiates SARS-CoV-2 infection. Furthermore, both pathogens may enter the systemic circulation for eventual entry through the blood brain barrier. The SARS-CoV-2 spike protein, in conjunction with CCL2, co-stimulates macrophages, resulting in IL-6 cytokine release. Likewise, Cpn infection leads to an increase of CCL2 and IL-6 cytokine release. The primary infection of either organism may lead to chronically elevated levels of IL-6 and secondary infection(s). Additionally, host APOEε4 expression appears to increase susceptibility to Cpn and SARS-CoV-2 infections.

**Discussion:**

Cpn and SARS-CoV-2 may enter the brain through olfactory neuroepithelial cells and/or through the blood brain barrier. SARS-CoV-2 utilizes specific receptors for infection, while Cpn utilizes binding of proteoglycans. Neuroinflammation may be an outcome of infection with one or both organisms as observed by increased levels of CCL2 and IL-6 leading to AD pathogenesis. Genetic risk is noted for infection with both organisms with expression of APOEε4. Ongoing and future studies will further dissect mechanisms of infection with SARS-CoV-2 and Cpn as they may inform on causation and diagnostic factors for AD.

## 1 Introduction

Alzheimer’s disease (AD) is the most common neurodegenerative disease worldwide, affecting approximately 55 million people (Yang et al., 2016). AD accounts for over 50% of dementia cases, characterized by a decline in intellect, memory, and personality that increases with age ending with death (Yang et al., 2016). Symptoms start with minor memory loss and can progress to severe cognitive decline and inability to perform essential daily tasks (Yang et al., 2016). AD is classified as either familial with mutational hereditary components that are autosomal dominant or as late-onset sporadic with both genetic and environmental factors contributing to disease (Czech et al., 2000; Wu et al., 2008).

The pathologic hallmarks of AD are beta amyloid (Aβ) plaques and tau neurofibrillary tangles which contribute to neurologic damage characteristic of AD. The beta amyloid precursor protein (βAPP) is a transmembrane protein found in various tissues, especially in the central nervous system. Under normal conditions, alpha secretase processes βAPP into harmless peptide fragments (Czech et al., 2000; Wu et al., 2008). However, in individuals with AD, βAPP is abnormally cleaved by beta and gamma secretase enzymes, producing Aβ peptides that aggregate to form Aβ plaques, a key feature of AD (Czech et al., 2000). Toxic Aβ peptides are thought to disrupt neuronal function thereby contributing to the memory loss and cognitive decline seen in AD patients (O’Brien and Wong, 2011). Tau proteins are microtubule-associated proteins that normally function to stabilize axonal microtubules. In AD, tau proteins become hyperphosphorylated, leading to the formation of harmful neurofibrillary tangles (Braak et al., 2006).

Despite extensive research, specific causation of AD remains unclear. However, one potential overarching hypothesis—the infection hypothesis—suggests that infectious agents, like bacteria and viruses (and potentially others) may trigger the neurodegeneration seen in AD (Itzhaki et al., 2004; Itzhaki et al., 2016). This neurodegeneration may lead to the onset of sporadic AD through multiple direct and indirect mechanisms such as neuroinflammation. Mechanisms may include direct invasion of the central nervous system (CNS) via cranial nerves such as through the olfactory neuroepithelium and trigeminal nerve, or indirectly by traversing the blood brain barrier (BBB) to invade the CNS possibly through the migration of leukocytes carrying infectious agents from the peripheral circulation (Dando et al., 2014).

A second related hypothesis, the antimicrobial protection hypothesis, further supports the infection hypothesis of AD. This hypothesis suggests that the brain’s response to these infectious agents is modulated by Aβ functioning as antimicrobial peptides (Moir et al., 2018). These antimicrobial peptides may be induced as an innate immune response against pathogens (Moir et al., 2018). In acute infections, antimicrobial peptides are neuroprotective by entrapment of pathogens, production of reactive oxygen species, and eradication of infected host cells. This suggests that Aβ can minimize the growth of pathogens such as gram-positive and gram-negative bacteria, certain fungi, and viral infections (Moir et al., 2018; Soscia et al., 2010).

Counterintuitively, excess antimicrobial peptides may dysregulate the oligomerization process and may lead to a more neurotoxic oligomeric species of Aβ. Although the antimicrobial protection hypothesis suggests that Aβ production can be a response to infection, it does not claim an infection is the only way that Aβ can be dysregulated. Other predisposing factors could precede infection to cause neurotoxic Aβ pathology (Moir et al., 2018). Thus, an infection could instead be a secondary insult rather than the sole initiating factor for AD pathology. In either scenario, the increased accumulating Aβ could be the result of the response to an infectious insult, potentially increasing the deleterious deposition of Aβ into plaques. Both primary infections and subsequent infection following AD initiation could both occur and are not mutually exclusive (Li et al., 2018). The combination of the infection hypothesis and the antimicrobial hypothesis encapsulates an understanding of late-onset sporadic AD progression with a potential bidirectional approach to chronic neuroinflammation with subsequent CNS neurodegeneration (Li et al., 2018).

Within this review, we highlight two particular infectious agents that may contribute to the pathogenesis process observed in late-onset sporadic AD. *Chlamydia pneumoniae* (Cpn), a respiratory obligate intracellular bacterium, and severe acute respiratory syndrome coronavirus 2 (SARS-CoV-2), a respiratory coronavirus responsible for the COVID-19 pandemic. Our review addresses peer-reviewed literature regarding the hypothesis that pathogens, specifically Cpn and SARS-CoV-2, may contribute to the development of AD due to their ability to initially infect the respiratory tract.

The recent pandemic of a respiratory disease which became associated with marked cognitive changes highlighted the need to study respiratory bacteria and viruses as contributing environmental risk factors for the development of further cognitive impairments. Our previous research with the respiratory pathogen Cpn and its association with AD (Balin et al., 1998; Gérard et al., 2006; Hammond et al., 2010) led us to also consider if the SARS-CoV-2 virus alone or in conjunction with this respiratory pathogen could contribute to an increased risk to develop AD. Given that Cpn is an obligate intracellular bacterium and SARS-CoV-2 is a virus, these two pathogens require cellular uptake to survive and/or reproduce. As they both can enter the nasal passages of the respiratory tract, they can infect the olfactory neuroepithelium, wherein they can bypass the BBB to infect and/or damage the olfactory structures in the CNS. Even more commonly, they can infect the airways of the lungs and subsequently have access to the pulmonary and systemic circulation potentially leading to eventual infiltration into the CNS through the BBB.

These routes of infection, via olfactory neuroepithelia and via the lungs into the blood, are considered as they allow for the potential of these airborne organisms to eventually impact the nervous system. We outline proposed mechanisms for pathogenicity and neuronal implications if infection in the brain is established. Further, we describe how infection in neuronal tissues can progress via the activation of inflammation and downstream metabolites.

Characteristics of these organisms are described and compared and contrasted with respect to how they may contribute to AD manifestation. Intriguingly, a viral hypothesis of AD has suggested that infections with other viruses such as herpes viruses, adenoviruses, polioviruses, etc. can lead to progressive CNS damage (Yong et al., 2021). Thus, we define factors and physiologic roles involved in Cpn and SARS-CoV-2 infections that may contribute to the progression of AD following an initial respiratory infection. During the pandemic, patients presenting with pneumonia symptoms were principally tested for SARS-CoV-2, whereas screening for SARS-CoV-2 in conjunction with bacterial co-infections was not prioritized. However, a large case study in the United States did report on co-infection of SARS-CoV-2 and Cpn (Richardson et al., 2020), while a few others also found co-infections with these organisms (Oliva et al., 2020; De Francesco et al., 2021).

Thus, although there is some consideration for co-infection evaluation, the major focus of this review is on the organisms as individual infectious agents with regard to how they may lead to an increased risk for the development of AD. Commonalities consistent with both infections are considered as major contributors to AD pathogenesis. As such, we propose the manner in which routes of infection, genetic predisposition and pro-inflammatory biomarkers may contribute to AD development and progression following infection with both Cpn and SARS-CoV-2.

## 2 Methods

Our review was undertaken with the knowledge that Cpn, being a respiratory pathogen, has been associated with AD; prior research has demonstrated Cpn’s presence in neuronal brain tissue (Balin et al., 1998; Gérard et al., 2006; Hammond et al., 2010). Further, SARS-CoV-2, another respiratory pathogen, has had an evolving association with reported neuronal symptoms during patient illnesses as the COVID-19 pandemic progressed (Rahman et al., 2021; Naughton et al., 2020; Matias-Guiu et al., 2023; Pang et al., 2024). Therefore, our review takes a closer look at each pathogen’s possible presence in the brain as an increased risk for AD development. The potential routes of each pathogen’s cellular entry were then researched and documented, as well as the associated enzymes and receptors that are involved in each entry. Both infection and receptors involved in pathogen entry were found to be modulated by certain genetic factors which were included in our review.

**FIGURE 1:**
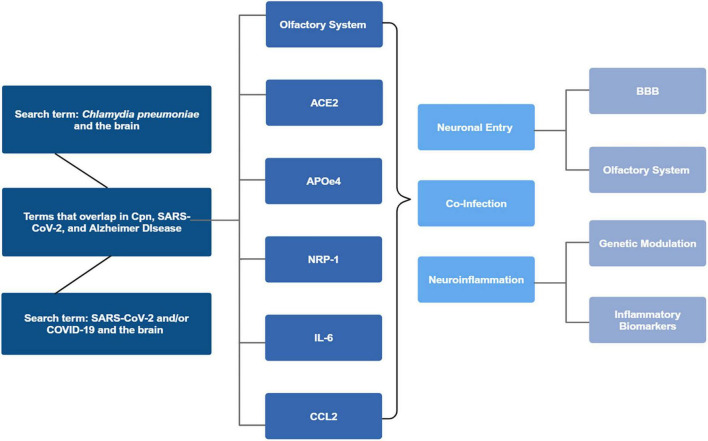
Search parameters flowchart. *Chlamydia pneumoniae* (Cpn) and the brain, COVID-19 and SARS-CoV-2 and the brain, and articles where the terms overlapped with Alzheimer’s disease were reviewed. Articles were identified for overlap with the olfactory system, ACE2, APOE status, NRP-1, IL-6, and lastly CCL2. Analysis of the articles revealed commonalities between the infectious processes and neuronal entry, neuroinflammation, co-infectivity and potential AD modulatory effects. Created in BioRender. Romanella (2025) https://BioRender.com/l7mlyv6.

Downstream biomarkers associated with Cpn were identified and then cross referenced with SARS-CoV-2 to determine similarities and differences between the microbes. These biomarkers were then researched in the context of AD to determine if they were disease modulators. We focused on specific biomarkers including: interleukin-6 (IL-6), chemokine ligand 2 (CCL2), and neuropilin-1 (NRP1). Additionally, the genetic risk factor apolipoprotein E allele ε4 (*APOE*ε*4*) was evaluated. Aspects of the infectious process were included, such as interaction of SARS-CoV-2 with the angiotensin-converting enzyme 2 (ACE2) receptor, which was particularly unique to viral entry. All of the above factors, in addition to neuronal inflammation were included in the analysis of infection in the pathogenesis of late-onset sporadic AD (see Figure 1).

## 3 Results

### 3.1 Intracellular respiratory organisms

#### 3.1.1 *Chlamydia pneumoniae (Cpn)*

Cpn is a gram negative obligate intracellular bacterium that mainly infects the respiratory tract but can also infect various organs and cell types throughout the body (Mackenzie, 2016). Once inhaled, Cpn can travel to the lungs and/or infect the nasal respiratory epithelium and olfactory neuroepithelium in the upper respiratory tract. The organism has a biphasic life cycle consisting of the infectious but relatively inactive elementary body (EB) phase and the mature, non-infectious reticulate body phase. As an obligate intracellular bacterium, it requires oxygen, ATP and other nutrients in order to survive and propagate within cells (Riffaud et al., 2023). In addition, Cpn is very effective in evading the normal immune system including the ability to infect monocytes (MacIntyre et al., 2002). Cpn can be detected in various biopsied or post-mortem tissues by immunolabeling with various anti-Cpn antibodies (Alberts et al., 2002).

**FIGURE 2:**
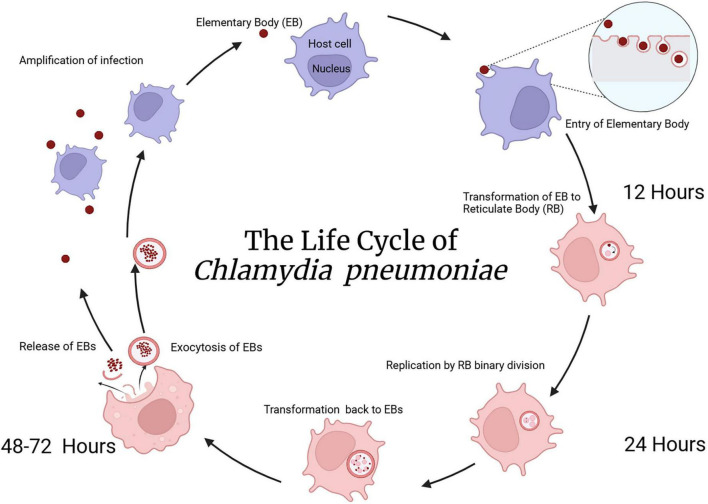
The life cycle of Cpn. As Cpn is an intracellular bacterium, initial uptake of elementary bodies into the eukaryotic cell proceeds through an endocytic mechanism. Following cellular entry, transformation from elementary to reticulate bodies occurs within a phagosomal inclusion. Replication of reticulate bodies in the phagocytic inclusion occurs over 24–48 h followed by transformation back to elementary bodies and exocytosis from the cell occurs after 48–72 h. This exocytosis of elementary bodies allows infection of other cells in the nearby vicinity. Created in BioRender. Romanella (2025) https://BioRender.com/hrya3ng.

Upon attachment to heparan sulfate proteoglycans on host cells (Moelleken and Hegemann, 2008), the EB is internalized through endocytosis, forming an intracellular inclusion. Inside this inclusion, the EB transforms into the larger reticulate body, which is metabolically active and undergoes replication using binary fission. After several rounds of replication, the reticulate bodies condense back into EBs which are subsequently exocytosed allowing for spread and infection of new cells thus completing the life cycle. This biphasic life cycle takes about 2–3 days for completion (Porritt and Crother, 2016; see Figure 2).

During times of stress, Cpn can enter a dormant/persistent state within the host cell, contributing to chronic infections that are difficult to treat (Panzetta et al., 2018). Further, Cpn’s ability to escape the body’s immune system allows it to remain in the cell for longer periods, potentiating prolonged inflammation. The organism’s unique life cycle plays an important role in the bacterium’s ability to sustain chronic infections, particularly in the respiratory and cardiovascular systems. Chronic infection triggers chronic inflammatory responses which result in severe cellular injury. This inflammation activates specific inflammatory mediators that contribute to oxidative stress, DNA damage, and altered cell repair mechanisms, creating a pro-inflammatory cycle. Repeated cycles of inflammation causing cellular damage leading to brain pathology may be responsible for the outcome of dementia and other neurocognitive disorders for which comparable cellular damage has been observed (Porritt and Crother, 2016).

#### 3.1.2 SARS-CoV-2

SARS-CoV-2 is an enveloped, positive sense, single stranded RNA virus (V’kovski et al., 2021) that is part of a subset of the Coronaviridae family known as beta-coronaviruses (Bai et al., 2022). Typically, viruses within the Coronaviridae family cause illnesses such as the “common cold,” with mild upper respiratory symptoms and illnesses that show up seasonally as the weather begins to get colder (V’kovski et al., 2021). There are three outliers to this family that cause highly pathogenic upper respiratory illness much more severe than the common cold: SARS-CoV, MERS-CoV, and most recently SARS-CoV-2 (V’kovski et al., 2021).

SARS-CoV-2 infects cells in order to propagate, essentially taking over the host cell machinery in order to survive. The genome of the virus codes for various protein templates necessary for viral replication. SARS-CoV-2 has 12 open reading frames which encode non-structural proteins (Bai et al., 2022). These non-structural proteins are required for viral RNA synthesis (Bai et al., 2022). SARS-CoV-2 also has four structural proteins: the spike protein, the envelope protein, the membrane protein, and the nucleocapsid protein (Bai et al., 2022). These proteins are all essential for entry and replication of the virus within the host cell.

As the COVID-19 epidemic continued to spread, there was increased recognition that mutations were occurring resulting in variants of the original virus leading to changes in pathogenicity, transmissibility and immunological responses (for review see Hattab et al., 2024). Viral variants included those from alpha to omicron and sub-variants with the spike protein undergoing mutations in the N-terminal and receptor-binding domains (Xia et al., 2020). The extent to which the original virus and variants cause respiratory symptoms and subsequent systemic symptoms are currently being addressed. There is evidence that following acute infection, and during post-infection monitoring, that cognitive symptoms appeared in those infected patients as compared to healthy controls (Crivelli et al., 2022). Investigations into the potential for SARS-CoV-2 to invade the nervous system leading to cognitive change have implicated both the blood brain barrier (BBB) and the intranasal olfactory system as possible routes of infection (Proust et al., 2023; Li et al., 2023).

### 3.2 Pathways for entry into the brain of intracellular respiratory pathogens

#### 3.2.1 Blood Brain Barrier

The BBB serves as a selectively permeable barrier between the blood and the brain. This barrier maintains homeostasis by allowing the exchange of ions, oxygen, carbon dioxide, glucose and other metabolites for typical brain function, while keeping foreign or toxic substances out. Many factors can disrupt the BBB and cause an increase in permeability; such as age and neuroinflammation (Knox et al., 2022). Functionally, the BBB consists of brain microvascular endothelial cells (BMECs) with tight junctions (TJ), and accessory cells such as pericytes, and glial cells such as astrocytes and microglia (for review see Yang et al., 2022b). The impermeability of the BBB is largely determined by junctional complexes interconnecting adjacent endothelial cells, including TJ and adherens junctions. TJ are composed of proteins such as claudin-5, occludins, and zonula occludins (ZO). These proteins primarily mediate the paracellular transport across the BBB (Stamatovic et al., 2016). Adherens junctions are formed by interactions between vascular endothelial cadherins, neural cadherins, and epithelial cadherins. These proteins adhere cells to one another and are associated with the signaling of ions in and out of the endothelial cells (Stamatovic et al., 2016).

Intriguingly, with regards to the blood brain barrier, a two-hit vascular hypothesis suggests that AD progression occurs through vascular damage and subsequent Aβ accumulation (Nelson et al., 2016). Cerebrovascular damage to the BBB alone can cause neurodegeneration via infiltration of substances not native to the brain, and also via diminishment in the clearance of neurotoxic substances, such as Aβ. This accumulation of neurotoxic substances contributes to the progression of AD (Nelson et al., 2016). The hypothesis also implicates a bidirectional relationship between BBB disruption and neuroinflammation (Takata et al., 2021), as BBB disruption could both lead to neuroinflammation directly, or be the result of chronic neuroinflammation.

Infiltration of SARS-CoV-2 and Cpn into the CNS could occur through an intact BBB following infection of leukocytes. This trojan horse scenario may occur in scenarios in which infected leukocytes traverse the BBB, such as during strokes, traumatic brain injury, or systemic infection. These scenarios may correlate to inflammation within the neuronal tissue and the further recruitment of potentially infected peripheral leukocytes to help repair and remove debris caused by the initial inflammation. Infected exogenous cells may proceed to activate astrocytes and microglia, exacerbating neuroinflammation and further disrupting the BBB. This disruption can then increase the permeability and infiltration of blood-borne substances, further amplifying glial activation and neuroinflammation in a positive feedback loop. In contrast, the BBB could be disrupted due to risk factors such as genetics, environment, and lifestyle factors (Nelson et al., 2016; Takata et al., 2021). Given this scenario, pathogens could infiltrate the CNS and subsequently trigger neuroinflammation.

SARS-CoV-2 infection can disrupt the BBB through endothelial cell infection and subsequent remodeling of TJ proteins. Yang and colleagues monitored the changes in TJ protein expression and organization *in vitro* with human BMECs and *in vivo* with BALB/c mice post-SARS-CoV-2 infection (Yang et al., 2022b). At 72 hours post-infection, there was a significant decrease in mRNA transcription levels of TJ proteins (Tight Junction Protein 1, occludins, and claudin-5) compared to control cells. This study also found the organization of these TJ proteins was irregular and scattered, suggesting a dysfunction of the BBB and increased permeability. These results suggest that SARS-CoV-2 through its spike protein can not only infect the BMECS, but also downregulate the expression of TJ proteins and modify their distribution (Yang et al., 2022b), thereby leading to disruption of the BBB with increased permeability and amplification of the inflammatory response.

Infected endothelial cells can also trigger a neuroinflammatory cascade within the brain by increasing mRNAs involved in processes such as inflammation, leukocyte migration, and angiogenesis (Yang et al., 2022b). Evidence for the inflammatory cascade and leukocyte migration suggests that infection of human BMECs elicits an immune response that can contribute to the inflammatory process. During an immune response, pro-inflammatory cytokines, such as IL-6, and chemokines, such as CCL2, are secreted to recruit immune cells to the site of infection to destroy any pathogens (Yang et al., 2022a,b). Excessive cytokine/chemokine secretion and increased immune cell reactivity can disrupt TJ and pericyte organization, thereby increasing permeability of the BBB to potentially allow both pathogens and leukocytes to enter the brain (Yang et al., 2022a). This process antagonizes the inflammatory response in the CNS and consequently can lead to neurodegeneration or worsening of existing neurological conditions such as AD.

Similarly, Cpn infection may also disrupt the BBB by damaging endothelial cells and altering the TJ arrangement, ultimately increasing permeability and promoting AD pathology (MacIntyre et al., 2002). Cpn can gain access to the CNS through the BBB from systemic infection and/or inflammation (Takata et al., 2021). One proposed mechanism of Cpn bypassing the BBB is following infection of monocytes within the vascular system with subsequent trafficking into the CNS. Another proposed mechanism is through direct infection of endothelial cells comprising the BBB (MacIntyre et al., 2002). MacIntyre and colleagues observed that human BMECs infected with Cpn *in vitro* increased adhesion molecule expression such as vascular cell adhesion molecule-1 and intercellular adhesion molecule-1 (MacIntyre et al., 2002). Similarly, Cpn infection of monocytes *in vitro* increased the expression of surface ligands, lymphocyte function-associated antigen-1 and very late activation antigen-4, integrins that bind to intercellular adhesion molecule-1 and vascular cell adhesion molecule-1, respectively. The combination of increased expression of adhesion molecules and ligands suggests a hyper-adhesive system, increasing the transmigration of Cpn-infected monocytes across the BBB model system. If Cpn activated monocytes enter the CNS, they could influence the activation of tissue-resident glial cells such as the microglia and astrocytes, causing an inflammatory signaling cascade (MacIntyre et al., 2002). Although the exact mechanism of how Cpn infiltrates the BBB and potentially activates glial cells is unknown, it is possible that lipopolysaccharide (LPS), an endotoxin released from gram-negative bacterial pathogens, including Cpn, is a contributing factor. In this regard, systemic infection or sepsis models demonstrate that gram-negative derived LPS can bind to pericytes and disrupt their arrangement around the microvessels of the BBB (Nishioku et al., 2009).

In addition to the BBB being a site of vulnerability for pathogen entry to the CNS, bypassing the BBB may also hold great importance for pathogen entry. This bypass mechanism is exemplified by respiratory pathogens that can directly infect the olfactory neuroepithelium in the upper respiratory tract. Entrance through this mechanism would effectively circumvent the brain’s protective BBB and enhance CNS spread of infection throughout olfactory connected regions of the brain.

#### 3.2.2 The olfactory system

**FIGURE 3:**
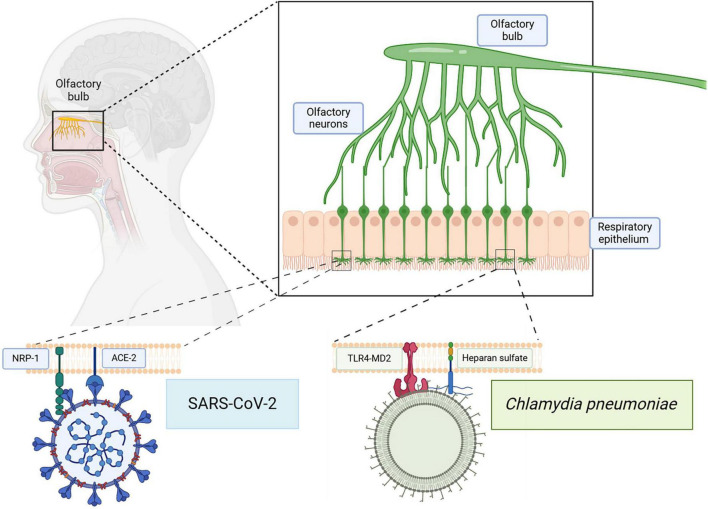
Infection of the olfactory system with SARS-CoV-2 and/or Cpn. The olfactory neuroepithelium is vulnerable to infections by respiratory pathogens. Receptors on these cells, such as NRP-1 and ACE2, can be utilized by SARS-CoV-2 for endocytosis. Other molecules such as Toll Like Receptor 4 (TLR4) and heparan sulfate proteoglycans may be used by Cpn for uptake. Direct infection via this pathway can bypass the blood brain barrier allowing access to other CNS structures. Created in BioRender. Romanella (2025) https://BioRender.com/hrvsh52.

In addition to the sense of smell, the olfactory system functions in the mapping of memory, place, context, emotion and reward. Various olfactory bulbectomy studies in animal models have demonstrated the impairment in spatial recognition, memory, and learning following olfactory bulb removal (Hozumi et al., 2003; Jaako-Movits and Zharkovsky, 2005; Nedogreeva et al., 2021). Olfactory bulb neuronal input and output projections suggest a deeper interplay between olfaction, memory, cognition, and learning. The olfactory bulb projects to various regions of the brain including amygdala, entorhinal cortex, hippocampus, and insula, allowing a point of entry for various respiratory pathogens and further spread into the CNS. Neuromodulatory areas projecting to the olfactory bulb include the locus coeruleus, basal forebrain, raphe nuclei, substantia nigra, and the ventral tegmentum (Johnson et al., 2000; Arnold et al., 2020). Together, the olfactory bulbectomy studies along with the olfactory bulb input and output projections demonstrate an interconnectedness between the olfactory system and cognitive functioning, learning, memory and emotion (see Figure 3).

One of the earliest symptoms of neurodegeneration, specifically in Parkinson’s disease and AD, is loss of olfaction (Ponsen et al., 2004; Woodward et al., 2017). Moreover, neurodegenerative hallmarks similar to that of AD are noted within the olfactory system. Post-mortem examination of brains affected by neurodegenerative disorders such as AD and Parkinson’s disease demonstrate, through histopathological analysis, pathological changes specifically in the olfactory bulb, including neuron loss and abnormal protein aggregates such as Aβ and alpha synuclein (Attems et al., 2014; Yoo et al., 2018; Arnold et al., 2010). Furthermore, pathological changes depicting neurodegeneration in AD and Parkinson’s disease begin in the olfactory bulb and appear far earlier than pathology in motor systems (Braak et al., 2003; Braak et al., 2006). This correlation of olfactory dysfunction as a prodromal finding of AD progression (Doty and Hawkes, 2019; Roberts et al., 2016; Christen-Zaech et al., 2003; Woodward et al., 2017) may potentially be utilized as a screening tool for AD (Devanand et al., 2015).

While olfactory dysfunction may occur in healthy, non-demented elderly patients, AD patients have significantly more severe olfactory glomeruli damage and Aβ protein accumulation compared to age matched controls (Son et al., 2022). Severe histopathological neurodegeneration in the olfactory glomeruli included alterations to neurotransmitter levels and neuronal size, shape, and density in AD patients as compared to controls (Son et al., 2022). Specifically, the olfactory glomeruli of AD patients demonstrated increased Aβ protein expression, a decrease in cell body size, and an increase in the number and size of microglial cells compared to age matched controls (Son et al., 2022). Additionally, the microglial cells in the olfactory glomeruli of AD patients demonstrated a morphological switch from a ramified shape, indicative of a resting state, to an amoeboid shape, indicative of an activated state (Son et al., 2022).

Magnetic resonance imaging has demonstrated functional impairment and significant structural atrophy of olfactory structures including the olfactory bulbs and cortical areas in AD patients compared with age matched controls. The progressive loss of volume of the olfactory bulbs and tracts observed correlated with disease progression from mild cognitive impairment to AD (Thomann et al., 2009). The distinction between peripheral and central olfactory structural damage using functional magnetic resonance imaging to observe brain activation in response to odor-visual association tasks was examined in three participant groups: cognitively normal controls, mild cognitive impairment, and those with AD (Vasavada et al., 2017). The study paired a visual cue with either an odor or odorless air. While the control group demonstrated significantly higher primary olfactory cortex functioning in the odor condition compared to the odorless condition, the mild cognitive impairment and AD group demonstrated reduced levels of primary olfactory cortex activity in both odor and odorless conditions. The comparable levels of reduced primary olfactory cortex activation in both odor and odorless AD groups indicate impaired central olfactory processing (Vasavada et al., 2017).

Additionally, positron emission tomography imaging studies demonstrated a correlation between increased cerebral amyloid deposition, olfactory dysfunction, and poorer cognitive performance among different stages of AD. Olfactory dysfunction can be indicative of amyloid accumulation and loss of cognitive function (Wang et al., 2023). Together, these studies suggest from structural, functional, and pathological findings that olfactory dysfunction can serve as a potential biomarker for the early diagnosis of AD.

Intriguingly, the nasal respiratory passages are vulnerable to infection with Cpn and SARS-CoV-2; subsequently, infection of the olfactory neuroepithelial cells and olfactory tissues will bypass the BBB. In this manner, the olfactory system can serve as a direct conduit for respiratory pathogens, such as Cpn and SARS-CoV-2, to enter into the CNS, impacting the sense of smell in the process (Bathini et al., 2024). As mentioned earlier, one of the first signs of neurodegeneration is loss of olfaction. Since SARS-CoV-2 is a respiratory virus, it often uses the respiratory epithelium to establish infection; however, it can also use the epithelium for olfaction that innervates the olfactory bulb (Hingorani et al., 2022), which would allow direct access to the brain bypassing the BBB. Upon further viral infection of neuronal cells, modulation of the neurologic-type of symptoms that we associate with COVID-19, such as loss of smell and brain fog, would be evident. Specific receptor recognition on olfactory neuroepithelial cells by SARS-CoV-2 is discussed below (see section 3.3.1). Since the late 1990s, Cpn has been associated with AD. In examination of post-mortem AD brains, Cpn was isolated and co-localized in regions with Aβ deposition and neurofibrillary tangles, including the olfactory bulbs (Balin et al., 1998; Gérard et al., 2006). Presence in the olfactory bulbs demonstrated that Cpn can infect the CNS through olfaction/olfactory bulbs (Chacko et al., 2022; Subedi et al., 2024). Furthermore, investigations by Little and colleagues demonstrated that intranasal delivery of Cpn to wild type BALB/c mice resulted in the organism’s presence in the olfactory bulbs and further in the brain along with identification of amyloid pathology (Little et al., 2005; Little et al., 2014). This was further validated by Chacko and colleagues who demonstrated in mice that Cpn enters the CNS through the olfactory and trigeminal nerves, thereby bypassing the BBB to cause neuroinflammation without initial hematogenous spread (Chacko et al., 2022). Chacko et al. demonstrated that Cpn can reach the mouse brain within 72 hours after intranasal inoculation, entering via the olfactory and trigeminal nerves (Chacko et al., 2022). Importantly, Chacko and colleagues found that Aβ deposits were found near Cpn inclusions, particularly in the olfactory nerve and bulb. Little and colleagues found similar depositions of Aβ amyloid in association with immunolabeling for Cpn (Little et al., 2005; Little et al., 2014). Aβ deposits were not found in adjacent tissue regions without Cpn inclusions (Chacko et al., 2022). Additionally, gene expression analysis showed that long term infection with Cpn leads to downregulation of genes involved in protein folding, thereby leading to protein misaggregation (Chacko et al., 2022). Moreover, as Aβ misfolding and deposition is a hallmark of AD pathology, this suggests that infection with Cpn results in neuroinvasion and pathology representative of AD (Chacko et al., 2022).

Interestingly, chronic inflammation in the olfactory neuroepithelium incites a functional shift of olfactory stem cells from regeneration to immune defense (Chen et al., 2019). Horizontal basal cells, the stem cells of the olfactory epithelium, promote the activation of T cells and macrophages via nuclear factor kappa B upregulation (Chen et al., 2019), therefore adding to an inflammatory cascade.

### 3.3 Receptors critical for infection

#### 3.3.1 ACE2

**FIGURE 4:**
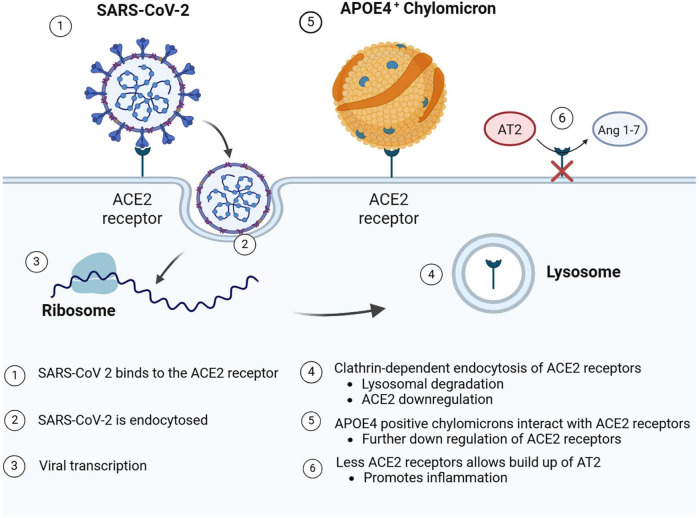
SARS-CoV-2 entry by binding to ACE2 receptors. Following receptor binding to ACE2 receptors on the cell surface, SARS-Cov-2 is endocytosed and released into the cytoplasm for viral transcription and replication. APOEε4 chylomicrons may also bind ACE2 receptors. Following either endocytosis of SARS-CoV-2 and/or APOEε4 chylomicrons, the ACE2 receptors may be down regulated or degraded. Such receptor down regulation may lead to the pro-inflammatory buildup of Ang 1–7. Created in BioRender. Romanella (2025) https://BioRender.com/m244l3i.

The cellular entry point for the SARS-CoV-2 virus is ACE2 (see Figure 4), which is an enzyme and receptor involved in the counter-regulation of the renin-angiotensin system. In normal physiological conditions, ACE2 allows for the conversion of angiotensin II (Ang II) to angiotensin 1–7, creating a favorable anti-inflammatory, anti-oxidant, anti-fibrosis, vasoprotective, and vasodilatory state (Haghighi et al., 2020; Chen et al., 2023). The SARS-CoV-2 virus uses ACE2 to enter the cells followed by spike protein priming (S1/S2 cleavage) accomplished by the cellular serine protease TMPRSS2 (Hoffmann et al., 2020). Following entry, there is downregulation of the ACE2 receptor on the cell’s surface (Chen et al., 2023), via lysosomal degradation through a clathrin-dependent mechanism (Lu et al., 2022). SARS-CoV-2 uses the respiratory epithelium to establish infection; however, it can also use the olfactory epithelium for entry to the olfactory bulbs (Hingorani et al., 2022). In epithelial cells of the respiratory tract, including the olfactory system, lungs, and oral cavity (Hingorani et al., 2022), the downregulation of ACE2 causes a build-up of Ang II, leading to a pro-inflammatory, pro-oxidant, pro-fibrosis state with vasoconstriction, and vascular leakage (Chen et al., 2023).

Viral infection of neuronal cells could modulate the neurologic-type symptoms that we associate with COVID-19, such as loss of smell and brain fog. A study by Wang and colleagues explored the susceptibility of neurons and astrocytes to SARS-CoV-2, demonstrating that not only could the virus enter and infect these cell types, but that infection could be modulated by certain genetic factors as well (Wang et al., 2021). SARS-CoV-2’s interaction with the ACE2 pathway may lead to an inflammatory response suggesting that whatever tissue may be infected, such as neuronal tissue once the virus passes the BBB, would potentially be at risk for an unchecked inflammatory cascade. Whether entry into the brain tissue and an increased inflammatory state could potentiate an early disease trajectory in an AD individual’s brain with other risk factors is of particular concern. Interestingly, there is evidence that even without a SARS-CoV-2 infection on board, lower levels of ACE2 have been found to be associated with Aβ and tau pathology found within AD brains (Kehoe et al., 2016). Kehoe and colleagues measured ACE2 levels within the mid-frontal cortex of human brain tissue after death, utilizing an AD group and a “non-demented” control group. Their results demonstrated that ACE2 levels were significantly less in the brains of the AD group compared to the control group (Kehoe et al., 2016). When evaluating specifically insoluble Aβ and tau pathologies, there was a significant inverse correlation (*p* < 0.01 for both pathologies) to ACE2, such that higher degrees of AD pathology were associated with lower levels of ACE2 expression (Kehoe et al., 2016). When an AD patient already expressing a down-regulation of ACE2, is then infected with SARS-CoV-2, a further downregulation of ACE2 as a result of infection, could result in accelerated AD progression.

#### 3.3.2 Neuropilin-1 (NRP-1)

NRP-1 is a cell surface receptor that plays a multisystem role in angiogenesis, tumor progression, and axonal guidance by acting as a receptor for ligands such as vascular endothelial growth factor-A, integrins, and transforming growth factor-β (Staton et al., 2007; Roskoski, 2007). Most relevantly, NRP-1 is abundantly expressed in respiratory and olfactory neuroepithelium and olfactory neurons (Zalpoor et al., 2022). NRP-1 can also be found in multiple tissue macrophages such as those in adipose tissue, alveoli, and bronchi. The transmembrane protein form of NRP-1 plays a key role in brain development, especially the hippocampus axonal projections (Bagri et al., 2003).

Due to the high expression of NRP-1 on the epithelial surface exposed to the external environment in the respiratory tract, SARS-CoV-2 is able to use this receptor as an entry point. The virus utilizes NPR-1 for cellular, vascular, and tissue penetration (Cantuti-Castelvetri et al., 2020). NRP-1 appears to potentiate the virus’ interaction with the ACE2 receptor, acting in synergy, and therefore increases the infection rate when compared to each receptor independently (Cantuti-Castelvetri et al., 2020). Expression of both NRP-1 and ACE2 in the olfactory tubercles may enhance SARS-CoV-2 entry, whereby the infection could further migrate centrally to reach the parolfactory gyrus (Zalpoor et al., 2022) and potentiate neurologic complications. Infection of astrocytes leading to reactive astrogliosis within brain tissue sets off a cascade of elevated inflammation and interferon production (Kong et al., 2022). This cellular response can cause damage to the neighboring neurons (Kong et al., 2022). Neuronal damage is likely to decrease acetylcholine production and therefore contribute to the further progression of AD (Kong et al., 2022) especially when considering the resultant neuroinflammation.

NRP-1 has been found to be expressed in higher amounts in AD brain tissues than in normal brains. High expression of NRP-1 in an AD patient may potentially increase the risk of an AD patient to be more susceptible to SARS-CoV-2 infection (Lim et al., 2021). A predisposition for SARS-CoV-2 due to high NRP-1 levels, combined with an *APOE*ε*4* positive status, could be the perfect storm for increased viral load, neuroinflammation, and subsequent cognitive decline observed in AD. In contrast, while extensive research has not directly linked Cpn with NRP-1 expression, a 2004 study by Shi and Tonunaga labeled Cpn infected cells for NRP-1 expression, yielding a positive correlation between the presence of the organism and NRP-1 (Shi and Tokunaga, 2004).

### 3.4 *APOE*ε*4* Vulnerability

A well-documented genetic modulator of AD is *APOE*ε*4*, coding for the APOEε4 protein, a member of a class of apolipoproteins present on chylomicrons that are involved in the metabolism of lipids as well as regulation of cholesterol transport (Chen et al., 2023; Wang et al., 2021). APOEε4 is one of many isoforms of APOE, and its expression confers the greatest risk for the development of AD (Farrer et al., 1997). While specifics as to how the *APOE*ε*4* allele leads to genetic risk for AD is not totally understood, both SARS-CoV-2 and Cpn may interact with the APOEε4 apolipoprotein to enhance their cellular infectivity.

APOEε4 has been previously studied in the context of Cpn infection in AD brains (Gérard et al., 2005). AD brain tissues were grouped according to their *APOE* allele type status (ε*2*, ε*3*,ε*4*) and the number of Cpn chromosomal copies in the brain tissues was determined with the use of PCR analysis (Gérard et al., 2005). Tissue samples from individuals who were *APOE*ε*4* positive had a significantly higher number of chromosomal copies of Cpn than the other allele groups, especially within the tissue samples taken from the hippocampus (Gérard et al., 2005). In a 2008 study of how *APOE*ε*4* may be modulating the increase in Cpn infection, Gérard and colleagues demonstrated via immunostaining, that the attachment of the Cpn EB to host cells expressing the *APOE*ε*4* allele was significantly higher than either cells expressing the *APOE*ε*3* allele or in cells lacking the predominant *APOE* alleles (Gérard et al., 2008). In fact, there were four times more infected cells in the *APOE*ε*4* culture as compared to the control cultures to suggest that the APOEε4 protein enhances the ability of the Cpn elementary body to bind to host cells. Increased infectivity of the cells and possibly tissues in which they reside could be a realistic outcome of the interactions between the APOEε4 protein and Cpn.

Recent studies demonstrate that *APOE*ε*4* may also be involved in an increased risk of infection with SARS-CoV-2 as well as the modulation of the ACE2 enzyme and receptor (Chen et al., 2023; Wang et al., 2021). Chen and colleagues first analyzed data from case-controlled studies to confirm the presence of a significant increase in infectivity and severity of disease in *APOE*ε*4* carriers; the results demonstrated significant increases for both. Follow-up to those results further demonstrated that the APOEε4 protein could bind directly to ACE2, and the spike protein of the virus could bind to the APOEε4 protein directly (Chen et al., 2023). Interestingly, in terms of binding, Chen and colleagues did not find any difference across APOE isoforms. When they shifted their focus to the ACE2 expression itself, they found that APOEε4 protein was down regulating ACE2 both *in vivo* and *in vitro*.

Wang and colleagues found a similar result when testing the SARS-CoV-2 infectivity of both neurons and astrocytes, specifically looking for differences between *APOE* genotypes. In terms of the number of infected cells, they observed more spike positive cells in *APOE*ε*4* neurons after 74 h post-infection than in *APOE*ε*3* neurons (Wang et al., 2021). Observations also included the presence of viral particles specifically in the dendrites of some of the neurons, as well as syncytia formation taking place preferentially in *APOE*ε*4* cultures than in *APOE*ε*3* cultures (Wang et al., 2021). In the 2021 study, when Wang and colleagues examined astrocytes, not only were there more spike positive cells in the *APOE*ε*4* astrocytes compared to *APOE*ε*3*, but the cell morphology differed between the two groups. *APOE*ε*4* astrocytes were found to have an increased size, as well as greater nuclei fragmentation, compared to *APOE*ε*3* astrocytes. Taken together, both the Chen and Wang studies demonstrated a possible mechanism by which an *APOE*ε4 genotype modulates SARS-CoV-2 infection (Chen et al., 2023; Wang et al., 2021).

### 3.5 Biomarkers of neuroinflammation

#### 3.5.1 Interleukin 6 (IL-6)

IL-6 is one of the pro-inflammatory cytokines released by the immune system in response to an infection, such as viral illnesses like COVID-19. During an immunological and inflammatory response, IL-6 can be secreted in two different manners: both T-cell independent and T-cell dependent secretion. Within the T-cell independent pathways, glial cells such as microglia and astrocytes are stimulated by an infectious agent and/or tumor necrosis factor α to release pro-inflammatory cytokines, such as IL-6. Once released, IL-6 plays a modulator role in the progression from acute phase inflammation to chronic inflammation (Kaur et al., 2020), by inducing continual microglial activation and recruiting new glial cells in the brain, leading to continued neuroinflammation (Khan et al., 2023). Microglia are especially important in chronic inflammation; they have been demonstrated to be chronically activated after a primary infection (Lull and Block, 2010; Millán Solano et al., 2023), and largely express IL-6 receptors (Haddick et al., 2017). As microglia and astrocytes are activated to contain infections, and thus, protect neurons from further damage, IL-6 released in response to inflammation could create a positive feedback mechanism contributing to this reactive gliosis (Lim et al., 2014).

In the instance of chronic neuroinflammation, the immune response becomes dysregulated leading to glial cell responses that target otherwise healthy neurons, leading to neurodegeneration (Millán Solano et al., 2023). Specifically, there seems to be an imbalance between the signaling of pro-inflammatory and inflammatory cytokines (Walker et al., 2019) from the neurotoxic (M1/A1) and neuroprotective (M2/A2) phenotypes of microglia and astrocytes (Kwon and Koh, 2020; Millán Solano et al., 2023). In considering AD specifically, there is evidence that microglia aid in the clearance of amyloid (Frautschy et al., 1992; Lim et al., 2014) suggesting that a change in function would cause amyloid accumulation. Signaling through the IL-6 cytokine also has a role in moderating the BBB (Khan et al., 2023), as IL-6 can increase BBB permeability by decreasing the expression of tight junctional proteins (Takata et al., 2021).

**FIGURE 5:**
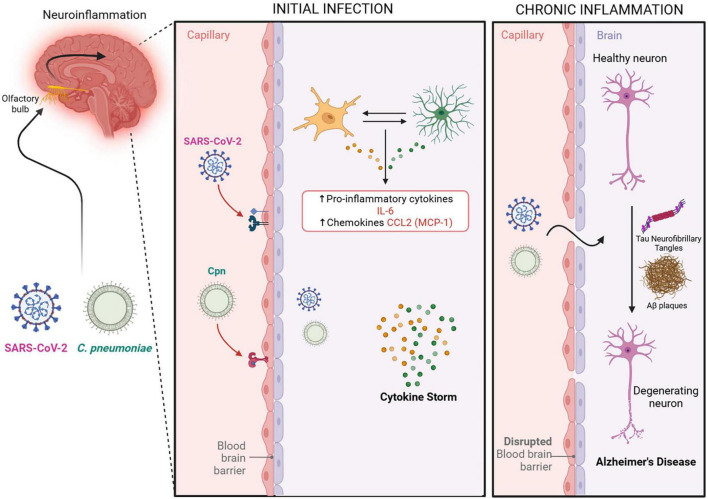
Potential neuroinflammatory outcomes following infection. An indirect pathway to the CNS by the pathogens is via the Blood Brain Barrier. SARS-CoV-2 and Cpn in the bloodstream may result in damage to and/or infection of the blood brain barrier allowing the passage of pathogens into the CNS. The compromised blood brain barrier may further result in acute and long term chronic neuroinflammation with increases in pro-inflammatory cytokines IL-6 and CCL2. Such chronic neuroinflammation may initiate or exacerbate the pathologies observed in Alzheimer’s disease. Created in BioRender. Romanella (2025) https://BioRender.com/fi5988q.

Intriguingly, SARS-CoV-2 infection following uptake by ACE2 and NRP-1 receptors via the olfactory system, can result in microglial cells becoming infected followed by their secretion of IL-6, thus amplifying neuroinflammation (Liu et al., 2023). SARS-CoV-2 astrocyte infection is also implicated in neuronal cell death (Crunfli et al., 2022; Liu et al., 2023). Further, an initial infection by SARS-CoV-2 may lead to chronic neuroinflammation and hyperphosphorylation of tau proteins (Pacheco-Herrero et al., 2021; Millán Solano et al., 2023). In severe cases of SARS-CoV-2 infection, systemic inflammation can increase BBB permeability, leading to astrocyte infection (Liu et al., 2023). Yang and colleagues found that SARS-CoV-2 infection of BBB endothelial cells induced high inflammatory responses with pro-inflammatory cytokine induction (Yang et al., 2022b). Further investigation has revealed that S1 subunits of SARS-CoV-2 variants can trigger IL-6 signaling in human brain endothelial cells with subsequent effects on microglia (Stangis et al., 2024). A large, unregulated production of pro-inflammatory cytokines, such as IL-6 by microglia and astrocytes, creates a cytokine storm that mediates a disruption in the BBB (Yang et al., 2022b). Along with infection, regardless of duration, a cofactor for decreasing BBB integrity would be aging (Liu et al., 2023). Thus, this decrease in BBB integrity can leave the elderly more vulnerable to further damage. The combined effects of infected astrocytes and microglia to cellular insult could explain the neurodegeneration and chronic inflammation, respectively, seen in the pathogenesis of AD (see Figure 5).

Infection with Cpn, as with other chlamydial species, will promote a pro-inflammatory cytokine response as a result of multiple factors such as heat shock protein expression and LPS presence (Rasmussen et al., 1997). Cpn invasion of CNS host cells (Krüll et al., 2005; Balin et al., 2018; Ma et al., 2022) may lead to a pro-inflammatory response to the Cpn’s LPS (Millán Solano et al., 2023). This response may contribute to the pathogenesis observed in AD (Balin et al., 2018), possibly by altering mitochondrial biogenesis genes resulting in amyloid plaque generation, tauopathy, and neuroinflammation (Millán Solano et al., 2023; see Figure 5).

Pro-inflammatory cytokines like IL-6 can ultimately influence cellular responses leading to outcomes correlating to AD pathology such as increased Aβ oligomerization and tau hyperphosphorylation (Walker et al., 2019). Cpn LPS can activate glial cells such as microglia and astrocytes, and in response to the infection, produce IL-6 to recruit even more glial cells to areas of infection (Boelen et al., 2007; Lim et al., 2014). Interestingly, *in vitro* murine experiments demonstrated that although Cpn infection was more prominent in astrocytes (>60%) as compared to microglia (<5%), the production of IL-6 was higher in microglia (Boelen et al., 2007). Nonetheless, cytokine assays of Cpn-infected murine microglia and astrocytes demonstrated that the concentrations of IL-6 for both were significantly elevated compared to controls at different time periods (Boelen et al., 2007). The secretion of IL-6 acts as a signal for other microglia and astrocytes to migrate to the site of infection, creating an additive production of inflammatory cytokines and potential neurotoxins. In the event of chronic inflammation, these cytokines and neurotoxins may not be cleared readily, and as a result, this cytokine storm may cause neuronal degeneration and cell death (Boelen et al., 2007). As mentioned previously, neuroinflammation induced by LPS can alter the BBB endothelial cells, increasing the BBB permeability and leukocyte infiltration (Lim et al., 2014; Millán Solano et al., 2023; Subedi et al., 2024).

#### 3.5.2 Chemokine ligand 2 (CCL2)

CCL2, also known as monocyte chemoattractant protein 1 (MCP-1), is a cytokine that acts as a chemoattractant for monocytes, T lymphocytes, and natural killer cells to sites of inflammation, tissue injury, or malignancy (Rollins, 1997; Evers et al., 2022). CCL2 is expressed in various tissues and cells, including monocytes/macrophages, astrocytes, microglia, endothelial cells, and neurons (Semple et al., 2010). Similarly, the CCL2 receptor (CCR2) is found in a diverse range of tissues including the olfactory nucleus, the limbic system, and outer cerebral cortices of the brain. Expression of CCR2 in these areas can be upregulated during states of inflammation (Banisadr et al., 2005). Olfactory damage, as exemplified with olfactory bulbectomy, or infection, may also lead to increased CCL2 levels, causing further transient upregulation of CCL2, CCR2, and CCR2 positive macrophages (Getchell et al., 2002; see Figure 5).

CCL2/CCR2 not only attracts immune cells, but also regulates the migration of monocytes through the vascular endothelia. CCL2 stimulation increases the elasticity and viscosity of the monocytes, thus improving their ability to migrate through junctions within the microvasculature (Evers et al., 2022). This has many implications for increased neuroinflammation, especially during times of infection when CCL2 levels may be increased. CCL2 has been associated with numerous neuroinflammatory and neurodegenerative disorders, including AD (Dimitrijevic et al., 2006; Hickman and El Khoury, 2010; Semple et al., 2010).

Within the CNS, CCR2 has been found to be expressed on brain endothelial cells, and stimulation of these receptors with CCL2 has been implicated in BBB changes. *In vivo* mouse studies found that intracerebral CCL2 injections lead to a decreased expression in TJ proteins (occludins, claudin-5, ZO-1, ZO-2) (Stamatovic et al., 2003; Stamatovic et al., 2005). Further, *in vitro* studies showed alterations in the BBB, specifically changes in TJ proteins, creating gaps between the endothelial cells following ischemic-reperfusion injuries. An upregulation of CCL2 and CCR2 was noted (Dimitrijevic et al., 2006). CCL2 appears to act both directly on endothelial cells by altering the proteins of tight junctions and indirectly by attracting monocytes which also change the permeability of the BBB.

Various studies have shown a duality in the downstream effects of CCL2. Although CCL2 plays an important role in inflammatory processes and can have a protective effect in various diseases, excessive levels can cause or worsen inflammatory conditions. In a similar manner, a reduction or inhibition of CCL2 can prevent neural damage but would lead to a diminished immune response. This suggests there is a delicate balance to CCL2 concentrations, and that deficiency can be as unfavorable as an excess (Gutiérrez et al., 2019; El Khoury et al., 2007; Semple et al., 2010).

Studies of monocytes infected with Cpn have shown a significant increase in CCL2, suggesting infection may lead to a pro-inflammatory state (Lim et al., 2014). Others have shown that macrophages and vascular smooth muscle infected by or exposed to Cpn release CCL2, which could cause vascular inflammation that can contribute to the development of atherosclerosis (Patil et al., 2016; Yang et al., 2005). Mice infected with Cpn show increased levels of CCL2 due to activation of the extracellular signal-regulated kinase (ERK) 1/2 pathway (Yang et al., 2005).

Similar to Cpn, SARS-CoV-2 infection can lead to an increased expression of pro-inflammatory cytokines by activating the ERK1/2 pathway (Ding et al., 2023; Yang et al., 2005). A significant increase in plasma CCL2 has been found in COVID-19 patients (Idiz et al., 2022). Additionally, CCL2 positive macrophages have been found to be in excess in bronchoalveolar lavage samples from patients with severe COVID-19 (Zhang et al., 2021). These higher levels of CCL2 correlate with increased severity and higher mortality in COVID-19 patients (Ding et al., 2023; Idiz et al., 2022). Ding and colleagues analyzed cytokine expression in macrophages exposed to SARS-CoV-2 antigens, specifically the spike protein (Ding et al., 2023). They found that macrophages co-stimulated with CCL2 and the spike protein had higher expression of the ERK1/2 pathway, and produced more pro-inflammatory cytokines (IL-6, CCL2, CCR2) than macrophages exposed to the spike protein or CCL2 alone (Ding et al., 2023).

CCL2, IL-6, and IL-8 (another pro-inflammatory mediator) have been found to be increased in AD brains and may play a significant role in the chronic inflammation that occurs in the CNS of these patients; specifically, within neurons, astrocytes, and plaques of AD brains (Sokolova et al., 2009). This increase was first demonstrated by analyzing the expression of cytokines and chemokines in homogenized AD brain specimens (Sokolova et al., 2009). These investigators found that CCL2 was elevated in all brain samples and the logistic linear regression showed CCL2 to be the most reliable predictor for AD, implying that it may play a significant role in the neuroinflammatory process of these patients (Sokolova et al., 2009). *In vivo* studies have shown that Aβ fragments [17-40,17-42, and 1-42], promote expression of CCL2 from differentiated human monocytes, and co-culturing with Aβ (1-40) promotes CCL2 production in human brain endothelial cells (El Khoury et al., 2003; Szczepanik et al., 2001; Vukic et al., 2009). Given the role of CCL2 as a chemoattractant for monocytes, as well as its effects on the BBB, an increase in CCL2 levels secondary to Aβ fragments likely allows for further monocyte infiltration. The role of CCL2 in AD is complex and, as mentioned previously, reflects CCL2’s physiologic impacts. Mice with CCR2 deficiency have been found to have impaired microglial accumulation and accelerated progression of neurodegeneration (El Khoury et al., 2007; Gutiérrez et al., 2019). However, mice that overexpress βAPP and CCL2 have been found to have CCL2-driven accumulations of monocyte-derived macrophages and microglia, as well as accumulation of Aβ (Yamamoto et al., 2005). These mice also display increased APOE protein levels, suggesting reduced Aβ clearance (Yamamoto et al., 2005). While CCL2 and CCR2 play a crucial role in the recruitment of microglia and removal of Aβ plaques to prevent progression of neuroinflammation, their overexpression also leads to neuroinflammation and plaque formation, suggesting that both overexpression and deficiency can have a damaging effect.

Thus, an infectious process leading to increased CCL2 levels could contribute to the neuroinflammation, neurodegeneration, and plaque formation seen in AD. These increased levels may also make the BBB more permeable allowing for even more of the infectious agent (Cpn or SARS-CoV-2) or pro-inflammatory cytokines and leukocytes to enter the brain. These influxes may lead to a cascade of further neuroinflammation and neurodegeneration.

## 4 Discussion

### 4.1 Infection with SARS-CoV-2 or Cpn may contribute to AD pathogenesis

**FIGURE 6:**
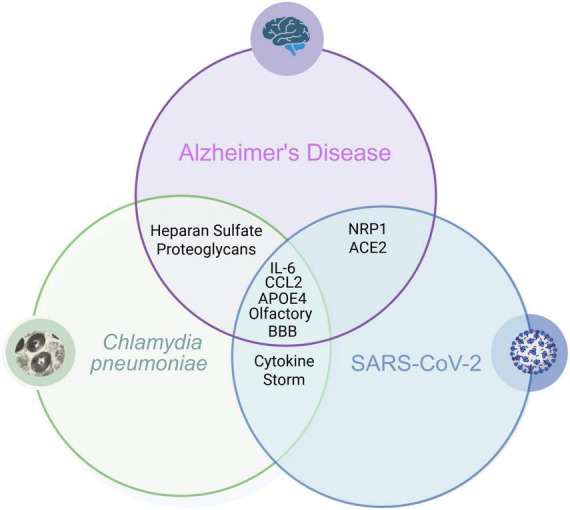
Overlapping biomarkers: Cpn, SARS-CoV-2 Infections and Alzheimer’s disease. Overlapping relationships demonstrate how infection with these organisms may be a risk factor for the development of Alzheimer’s disease. Created in BioRender. Romanella (2025) https://BioRender.com/qwrsnij.

While the exact pathology of AD is unclear, there is evidence to suggest that its development may be linked to inflammation of neuronal tissue, specifically in the limbic system. There are many situations that can cause neuroinflammation, one being infection with microbes that are able to gain entrance to the CNS. We have described a few pathways by which Cpn and SARS-CoV-2 can gain entry to the CNS and proceed to promote inflammation via modulation of an inflammatory cascade. We therefore propose that in certain settings, such as with aging and genetic predisposition, that infection with these pathogens may be a risk factor for the development or modulation of AD. Longitudinal neuronal monitoring with cognitive function testing should be added to studies to determine long term outcomes of microbial infection within the realm of neurologic dysfunction. Even more, pathology analysis should also focus on infection identification in peripheral tissues in living patients and within the CNS at autopsy (Lathe et al., 2023). Therefore, finding reliable biomarkers may be the key to determining if these and other microbes are able to chronically inhabit neuronal tissue after initial infection. Applicable outpatient testing options may be to test for biomarkers of previous/current infections or antibody signatures present within blood, saliva, urine and even nasal swabs. When these results are coupled with consistent cognitive function testing, and testing for Aβ and tau biomarkers for AD pathology, we may be able to better understand how pathogens may be contributing to the development of AD (see Figure 6).

Given the mechanisms of infection by both SARS-CoV-2 and Cpn, the possibility that a long-term co-infection could exacerbate the neuroinflammation seen individually for each organism could be likely. Additionally, the similarity of infections and their immune responses could be synergistic, in that, immune cells that are activated to react to one pathogen may effectively react to the other. More studies are needed to confirm the relationship between SARS-CoV-2 and Cpn co-infection and chronic inflammation.

Screening for SARS-CoV-2 in conjunction with bacterial co-infections was not prioritized during the COVID-19 pandemic, but some investigations were carried out and have reported an association of Cpn and *Mycoplasma pneumoniae* co-infection along with or following a SARS-CoV-2 infection (Oliva et al., 2020; De Francesco et al., 2021). Although, it was noted that the evidence of a co-infection of SARS-CoV-2 and Cpn was less than that of SARS-CoV-2 and *Mycoplasma pneumoniae* (Frutos et al., 2022). A large in hospital case study in the United States only reported 2 out of 5,700 patients with a co-infection of SARS-CoV-2 and Cpn (Richardson et al., 2020) while one by Olivia and colleagues, reported 5 out of 182 (2.7%) co-infections of both organisms (Oliva et al., 2020). Still, another study by De Francesco and colleagues reported 197/433 (40.41%) co-infections with both organisms (De Francesco et al., 2021). A possible explanation for the difference in co-infections is a retrospective analysis of data during a time when screening patients, who were positive for SARS-CoV-2 for other pathogens, was not a priority. The lack of screening for these respiratory bacterial infections may also be due to cross-over symptomatology. Another correlation observed in studies was between presence of co-infection and the severity of symptoms. Olivia and colleagues reported only one patient needing ICU intervention and no deaths (Oliva et al., 2020). De Francesco and colleagues reported the number of patients in critical care with a co-infection of SARS-CoV-2 alongside Cpn and/or *Mycoplasma pneumoniae* was significantly higher than those only infected with SARS-CoV-2 (De Francesco et al., 2021). Worse respiratory and radiological features and more intensive care admissions were noted with individuals who had co-infections with Cpn and *Mycoplasma pneumoniae* and SARS-CoV-2 (Guarino et al., 2022). Although, even with these features, there was no increase in mortality rate with these co-infections (Guarino et al., 2022). With co-infections of other organisms including viruses, fungi and other bacteria, severity of disease could be driven by the extent of the pro-inflammatory response leading potentially to a cytokine storm, increased coagulopathy and myocardial injury (Baral et al., 2024; Chang et al., 2025). Similar to the reported cases of co-infection, the data on the relationship between co-infection and the necessity for critical intervention were inconclusive. Additionally, an interesting result post-vaccination for SARS-CoV-2 was a changing pattern of bacterial co-infections with gram-negative organisms (decline) suggesting a co-protection evolved among the vaccine, virus and bacterial interplay (Said et al., 2024).

### 4.2 Infection of the olfactory system and the BBB

SARS-CoV-2 appears to function very similarly to the intracellular bacterium, Cpn, in that it can evade the immune system by hiding inside normally appearing cells and potentially enter the brain contributing to neuroinflammation. This feature may also be responsible for the ability of both the virus and bacterium to potentially traffic into brain tissues following infection of the circulatory system or following direct infection of peripheral olfactory tissues leading to the brain. Once inside the brain, the presence of the organisms may activate glial cells, thereby initiating an inflammatory cascade within the neuronal tissue. Both pathogens are also capable of infecting the endothelial cells of the BBB to modulate its functions, allowing for increased permeability. SARS-CoV-2 infection may result in decreased TJ organization making the barrier leakier (Yang et al., 2022b), whereas Cpn appears to create a hyper-adhesive environment that may alter the expression of TJ proteins whereby infected monocytes could readily be trafficked across the BBB (MacIntyre et al., 2002). Wang’s findings of viral particles in neuronal dendrites as well as the development of syncytia could suggest possible routes of viral spread within the brain if the infectious agents were to make their way into the olfactory bulb or through the blood brain barrier. Furthermore, a leaky BBB would allow for other microbes and toxins to make their way into neural tissue; special attention to this outcome should also be considered as additional inciting agents may contribute to the overall pathogenesis of AD (Bathini et al., 2024).

The olfactory system, on the other hand, provides a direct pathway for respiratory pathogens to invade olfactory structures such as the olfactory bulb as well as gain further access to the amygdala, entorhinal cortex and hippocampus in the CNS. As the olfactory system has been implicated in entry for both SARS-CoV-2 and Cpn infections (Bathini et al., 2024), and as olfaction communicates directly with the limbic system, infection in this system could result in inflammation, damage, and pathology implicated in AD. Studies have shown that when the olfactory bulb is removed, it alters memory and cognition (Hozumi et al., 2003; Jaako-Movits and Zharkovsky, 2005; Nedogreeva et al., 2021). The olfactory bulb could be postulated as a nidus for AD if damaged, as much of the AD pathology recognized has been seen to first appear in the olfactory bulb and subsequent brain areas such as the lateral entorhinal cortex (Attems et al., 2014; Yoo et al., 2018; Arnold et al., 2010).

More specifically, Cpn was co-localized with AD pathologic features (Aβ amyloid) in relevant brain regions including the olfactory bulbs (Balin et al., 1998; Gérard et al., 2006; Hammond et al., 2010). This supports the findings that Aβ may function in immune defense as an antimicrobial peptide in response to infectious agents (Moir et al., 2018). It follows, therefore, that pathogen invasion of the CNS whether through the BBB, or bypassing the BBB via the olfactory system, could result in Aβ aggregation and deposition following processing of the βAPP into relevant Aβ peptide species. As Cpn may also be able to survive long term in glial cells as a persistent infection, chronic inflammation may be generated leading to ever increasing pathology expanding within broader CNS regions.

Future research should continue to focus on infection as an initiator of the early inflammatory insult in AD, beginning with olfactory infection and spreading via the various limbic system connections. Furthermore, better screening modalities for SARS-CoV-2 and Cpn and/or biomarkers consistent with these infections within pathologic tissue samples may be necessary to unequivocally demonstrate their involvement in AD pathogenesis.

### 4.3 Neuroinflammation and AD

Exposure to infectious agents leads to an initial immune response that attempts to remove the pathogens before infection or cellular damage occurs (see Figure 5). The response is tailored to the infecting pathogen but generally leads to activation of immune cells, production of cytokines/chemokines, recruitment of immune cells, and adaptive responses such as the production of antibodies to eliminate the infectious agent and infected cells (Chaplin, 2010). Infection by SARS-CoV-2 or Cpn will elicit an immune response that may cause dysfunction of the BBB and/or the olfactory neuroepithelium, allowing for pro-inflammatory immune cells and molecules to spread into the brain parenchyma. Several studies have provided evidence of pathogens within the brain of AD patients, one being the initial Cpn publication by Balin and colleagues in 1998 (Balin et al., 1998). The studies have investigated a diverse array of viruses, bacteria, fungi, and protozoa (for review see Itzhaki et al., 2016; Lathe et al., 2023). Of interest, a number of the pathogens associated with AD pathogenesis are intracellular organisms. For example, the 2017 review by Sochocka and colleagues on the infectious etiology of AD noted 19 different infectious agents, all facultative or obligate intracellular organisms, linked to the development of AD (Sochocka et al., 2017). This can be due to intracellular organisms’ ability to evade host immune responses, allowing them to create chronic, persistent, and/or latent infections which may act as a nidus for chronic neuroinflammation that has been implicated in late onset AD.

### 4.4 SARS-CoV-2 and Cpn interactions with *APOE*ε*4*

The *APOE*ε*4* genotype has been shown to be a significant risk for AD as well as a modulating factor for many diseases across a variety of bodily systems, such as atherosclerosis in the cardiovascular system and infection systemically. With regards to infectious disease, APOEε4 protein may be involved in the uptake of organisms into host cells. This is especially important for Cpn and SARS-CoV-2 which need to be intracellular for their replication and survival. From this aspect, the pathogens would be able to enter cells of neuronal tissue as a function of the patient’s *APOE*ε*4* positive genetic status contributing to their overall virulence.

Intriguingly, there appears to be a relationship between APOEe4, HSPGs, AD, and risk for infection with both SARS-CoV-2 and Cpn. Previous studies have identified that enzymatic expression of HS3ST2, an enzyme leading to heparan sulfate synthesis, is involved in abnormal phosphorylation of the tau protein (Sepulveda-Diaz et al., 2015) and that HSPGs are involved in the neuronal internalization, propagation and transsynaptic transmission of tau (Holmes et al., 2013; Rauch et al., 2018). Furthermore, astrocyte-secreted glypican 4, a type of HSPG, has been shown to strongly interact with APOEe4 to increase APOEe4-induced tau hyperphosphorylation (Saroja et al., 2022). Clinically, the evidence implicating HSPG directly with AD has become more apparent as an analysis of two large US health systems’ electronic health records revealed that treatment with heparin was associated with a delayed diagnosis of AD (Readhead et al., 2024). With the association of APOEe4 with both SARS-CoV-2 and Cpn infection and AD, and with HSPG involved with organism uptake as well as directly involved with AD tau pathology, a complex interconnected relationship becomes more apparent with consideration of infectious involvement in disease pathogenesis.

With regards to SARS-CoV-2, the APOEε4 protein was shown to interact with ACE2, specifically downregulating the enzyme. The reason as to why this mechanism of action of APOEε4 downregulation of ACE2 is so important is that when it is coupled with SARS-CoV-2’s inherent ability to also down regulate ACE2, one could postulate a synergistic effect. This could mean an aggressive increase in downregulation of ACE2 levels which ultimately leads to inflammation due to inability to break down Ang II. The implications for AD come from the evidence on decreased levels of ACE2 found in autopsy brain tissue from AD versus healthy cadavers. Combining this, a severe decrease in ACE2 from a SARS-CoV-2 infection on a host *APOE*ε*4* genetic background could be setting the brain up for rapid AD modulation or development. When looking at infectivity, *APOE*ε*4* positive cells were infected more readily and had higher amounts of spike positive cells than *APOE*ε*3* counterparts (Chen et al., 2023). There was also syncytia formation that was noted specifically within the *APOE*ε*4* cell groups. This could be important particularly when viewing an infection within the olfactory bulb, as syncytia formation could allow for viral spread easily to other areas of the limbic system in the brain.

A possible mechanism for increased neuronal inflammation in *APOE*ε*4* cells could be as follows based on the research that we have collected; first, *APOE*ε*4* positive cells are more readily infected, and those cells create more inflammation as compared to *APOE*ε*4* negative cells, due to the proteins ability to downregulate the ACE2 enzyme. Then, these infected cells go through a more severe cytopathogenic process than *APOE*ε*4* negative cells, which may further contribute to an inflammatory response in the neuronal tissue. Overall, these infected cells may create a nidus for continued inflammation.

While the exact mechanism that causes this increased infection rate of cells by both organisms remains unclear, future studies should explore how *APOE*ε*4* genotype cells as well as the APOEε4 protein are involved in the interaction between these microbes, and others such as HSV1, and host cells. Studies should also look into why cells that are *APOE*ε*4* positive and infected with SARS-CoV-2 are more compromised as compared to their *APOE*ε*4* negative counterparts.

### 4.5 Limitations and future directions

Our review focuses on two infectious agents that may be important in the pathogenesis of sporadic late-onset AD. While accumulating data has demonstrated multiple associations and potential mechanisms by which infectious agents are associated with this neurodegenerative condition (Bathini et al., 2024), proof of infectious causation requires continual in-depth analyses of *in vivo* and *in vitro* studies. Considerations in these investigations should include more extensive diagnostic approaches with regards to presence of microbial agents that may be contributing factors. Greater consensus in these approaches may uncover important, yet to be totally accepted relationships, between infection and neurodegenerative disease (Lathe et al., 2023).

In this regard, as SARS-CoV-2 is a relatively new virus with multiple variants, there is a great need for further understanding of long-term effects on the body and specifically the brain. As the virus has been found, at times, to be able to enter into the CNS, the long-term effects—as exemplified by neurocognitive change and the brain fog seen in the long-haul cases—are unknown. Some studies have indicated that deficits correlating to COVID-19 include those of memory impairment, attention, executive functioning and language (Matias-Guiu et al., 2023). Association of these deficits with inflammation and inflammatory profiles has been and is currently being investigated (He et al., 2023). Long-term neurocognitive impacts of disease are being evaluated to explore issues such as immune system involvement, inflammatory responses, and viral neurotropism (Pang et al., 2024; Panagea et al., 2025). Although this review has discussed the ability of the virus to initiate neuroinflammation, as of yet we do not know the full extent of the resultant damage from this neuroinflammation nor the biomarkers that may reflect such long-term damage. Analysis of whether a SARS-CoV-2 reservoir might exist in some individuals contributing to chronic disease and long COVID is currently underway with considerations of reservoirs in tissue sites including the heart, lungs, brain and musculoskeletal systems (Proal et al., 2025).

While SARS-CoV-2 identification, studies, and treatments have been primarily focused on the singular presence of one virus, coinfection studies have been limited. Potential polymicrobial contributions to the COVID-19 pandemic and subsequent disease and development of long-term chronic issues such as Alzheimer’s disease needs further study. The contributions of co-infections may accelerate or exacerbate the contribution of a single organism alone. The overlap of cellular responses of Cpn and SARS-CoV-2 suggests the possibility that individual and/or polymicrobial infections may have compounding effects and warrants more study.

A further limitation that we recognized during our research concerning co-infections is that individuals presenting at the emergency department or who are admitted to the hospital with a respiratory infection or community acquired pneumonia are rarely tested for all causative organisms, and are rather treated empirically. As many hospitals still have protocols in place for testing for SARS-CoV-2, the numbers of SARS-CoV-2 cases may be more accurate for that virus alone. Due to the lack of testing, the true number of both Cpn caused pneumonia and pneumonia due to co-infection of Cpn and SARS-CoV-2 may be under reported.

With the potential for stealthy infectious agents infecting an individual, analysis of polymicrobial contributions to downstream CNS disease(s) is warranted. The effects of the SARS-CoV-2 virus on the CNS may be exacerbated by the presence of other organisms, including those of the gut microbiota, making the CNS even more vulnerable to pathogen influence. Further, initial CNS infections by other organisms may in turn leave the CNS more vulnerable to SARS-CoV-2 and its effects. Thus, expanding rapid detection with respiratory pathogen panels and/or analysis of multiple bodily fluids to determine all the organisms on board may give us more information. Identifying the polymicrobial contributions to downstream effects may inform interventions leading to amelioration of CNS damage.

Another limitation is the lack of information related to the genetic background of the infected individuals. As noted in the review, individuals with an *APOE*ε*4* genotype appear to be more vulnerable to infections; other genetic vulnerabilities (HLA type, ABO blood factors, immune suppression, etc.) will need to be considered in future studies. Furthermore, other chronic conditions such as diabetes or cardiovascular disease for which infection may or may not be directly involved need to be addressed as these comorbidities could likely increase the risk of infection-associated CNS disease.

Lastly, sex differences have been identified in the development of AD. In parallel, the sex differences seem to have a relationship with the vulnerability of people to certain infections and immune dysregulation. Thus, the underlying vulnerabilities to both infections in this context and downstream CNS damage require further study.
